# Drug Therapies for COPD: A Bibliometric Review From 1980 to 2021

**DOI:** 10.3389/fphar.2022.820086

**Published:** 2022-04-20

**Authors:** Gao Zhen, Liu Yingying, Dong Jingcheng

**Affiliations:** ^1^ Department of Integrated Traditional Chinese and Western Medicine, Huashan Hospital Affiliated to Fudan University, Shanghai, China; ^2^ Department of Retired Veteran Cadres, Putuo Hospital, Shanghai University of Traditional Chinese Medicine, Shanghai, China

**Keywords:** COPD, medications, bibliometric analysis, pulmonary drug delivery systems, elderly

## Abstract

**Objective:** To analyze all chronic obstructive pulmonary disease (COPD) drugs-related articles that were indexed in the Web of Science Core Collection (WOSCC) database until August 28, 2021 using bibliometric analysis, in order to provide a reliable reference for the treatment of COPD.

**Methods:** A comprehensive search was conducted to analyze all COPD drugs-related articles using WOSCC database from inception to August 28, 2021. Abstracts and potentially eligible articles, which were retrieved during literature search, were screened by two reviewers. Besides, the CiteSpace (5.8.R1) software was utilized to analyze the overall structure of the network, the network clusters, the links between clusters, the key nodes or pivot points, and the pathways.

**Results:** A total of 2552 COPD-drugs related articles were retrieved. From the perspective of categorization of published articles based on country, the United States is the country with the largest number of published articles and completed clinical trials, highlighting the important role of this country in the treatment of COPD. However, in terms of the proportion of ongoing clinical trials, China has the highest proportion, suggesting that China will play a more pivotal role in the medication of COPD in the future. From the perspective of cooperation among countries, the cooperation among European countries was closer than that among Asian countries. In the recent three decades, the top 20 institutions, with a particular concentration on the treatment of COPD, were from North America and Europe. The co-citation analysis showed that, among 2,552 articles, 53154 citations were recorded, and the co-citation network indicated that 24 clusters could be achieved.

**Conclusion:** The administration of bronchodilators and pulmonary drug delivery systems, as well as consideration of elderly COPD patients remained the hotspots, while triple therapy and comorbidity of COPD, as well as the prevention and treatment of elderly COPD patients had been frontiers in recent years.

## 1 Introduction

Chronic obstructive pulmonary disease (COPD) is a common, preventable and treatable disease, characterized by persistent airflow limitation that is mainly progressive and is associated with an enhanced inflammatory response in the airways and lung to noxious particles or gases (GOLD, 2021). The pathology of COPD encompasses a variety of structural alterations, involving airways, lung parenchyma, and pulmonary vasculature (Decramer et al., 2012). An epidemiological survey showed that the prevalence of COPD among Chinese adults and smokers who aged >40 and >60 years old was 13.7% and 40%, respectively (Wang et al., 2018). A survey performed in South Korea revealed that the prevalence of COPD among adult non-smokers who aged ≥40 years old was 6.67% (Kim et al., 2014). COPD has gradually become a global public health crisis (Patel et al., 2019). The World Health Organization (WHO) announced that the prevalence of COPD will continue to increase in the upcoming 40 years, exceeding 5.4 million COPD and other related diseases patients who will annually die by 2060 (Chronic Obstructive Pulmonary Disease Group of Chinese, 2021). COPD generates substantial costs for the health system, mainly related to moderate to severe stages and the exacerbations and complications entailed, (Gutiérrez Villegas et al., 2021) and the biggest driver of these healthcare costs is hospitalization (Khakban et al., 2017). Thus, how to prevent or delay the progression of COPD and reduce the frequency of acute exacerbations not only improves patients’ quality of life and reduces mortality, but also saves medical costs. At present, traditional Chinese medicine, such as Tai Chi and Qigong, may be significant for the treatment of COPD patients in terms of enhancing lung function, relieving dyspnea, and improving patients’ quality of life (Ding et al., 2014). However, the most important treatment for chronic obstructive pulmonary disease is still to give patients the correct drug treatment through various channels. The current treatment of COPD is mainly based on different combinations of bronchodilators and inhaled corticosteroids, and the amount of drug have increased during the exacerbation of COPD. However, the therapeutic effect is sometimes not ideal, and the long-term use of some drugs will produce side effects with different manifestations. In recent years, with the gradual deepening of the etiology, pathogenesis and clinical research of COPD, the development of new drugs for the treatment of COPD has become possible. Bibliometrics is a statistical method which could quantitative analysis the research papers concerned about one special topic via mathematical ways (Chen et al., 2014). It could also access the quality of the studies, analysis the key areas of researches and predict the direction of future studies (Yu et al., 2020).

In this study, we employed bibliometric analysis to analyze all COPD drugs-related articles that were indexed in the Web of Science Core Collection (WOSCC) database until August 28, 2021, in order to provide a reliable reference for the treatment of COPD.

## 2 Methods

### 2.1 Search Strategy

A comprehensive search was conducted to analyze all COPD drugs-related articles using WOSCC database from inception to August 28, 2021. Abstracts and potentially eligible articles, which were retrieved during literature search, were screened by two reviewers. Any discrepancies between reviewers in the study selection were resolved via consultation with a third reviewer. Those articles that entitled the terms “COPD” or “chronic obstructive pulmonary disease” or “chronic obstructive pulmonary diseases” were included in our search. The search indices included Science Citation Index Expanded (SCI-Expanded), the Conference Proceedings Citation Index–Science (CPCI-S), and Current Chemical Reactions Expanded (CCR-Expanded). WOS Core Collection (formerly Institute for Scientific Information Web of Knowledge) is the most used and authoritative research literature search engine, providing comprehensive coverage of key research outputs from around the world. It is a multidisciplinary database with more than 100 subjects, including the major sciences, arts, humanities, and social sciences (e.g., political science, architecture, and philosophy) (Shao et al., 2021).

### 2.2 Data Extraction and Quality Assessment

Data extraction and quality evaluation were performed independently. After searching in the WOSCC database, the number of publications and the total and average citations for the authors and journals were recorded. For authors actively publishing on COPD-related drugs, the following indicators were measured: the h-index, which is the number of publications and the number of times the publication is cited; the R-index, which is the square-root of the total citation frequency in the h-core, defined by the h-index; the h^(2)^-index, or the number of publications h^(2)^ that are cited at least [h^(2)^]^2^ times; and the i10-index, or number of publications cited at least 10 times. The higher the values of these bibliometric indicators, the greater the influence of the authors and their publications. Besides, the CiteSpace (Chen, 2006) (5.8.R1) software and VOSviewer (van Eck and Waltman, 2010) (1.6.17) was utilized to analyze the overall structure of the network, the network clusters, the links between clusters, the key nodes or pivot points, and the pathways. A node in the map represented the type of study being analyzed, and links between the nodes represented relationships or collaborations, co-occurrence, or co-citations. For literature analysis, the time slice was 1 year, and the correlation strength was cosine. The threshold for each time slice selected Top N = 50.

### 2.3 Number of Clinical Trials

Using the name of the country as the search term, the COPD clinical trials carried out in various countries since the establishment of the database were searched in the American clinical trials database (https://clinicaltrials.gov).

## 3 Results

### 3.1 General Data

**FIGURE 1:**
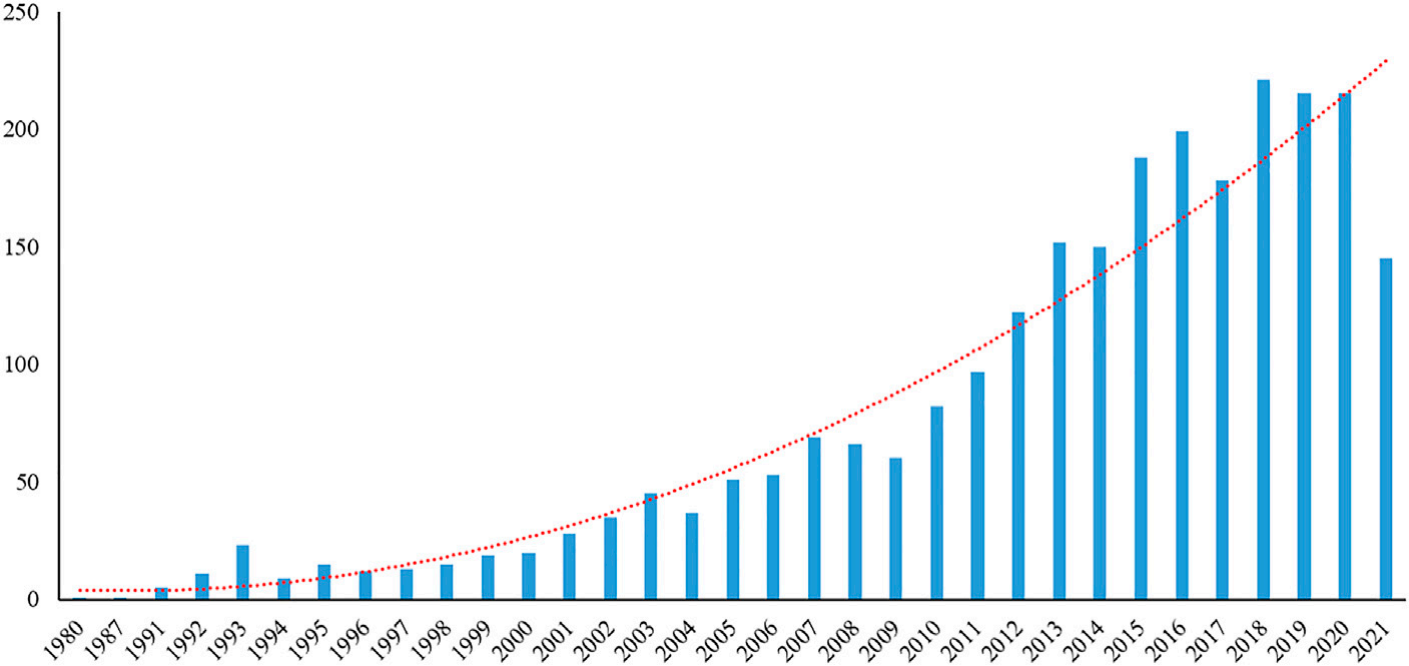
The statistics of COPD drugs-related articles from 1980 to 2021.

From 1980 to August 28, 2021, 2552 articles were published. From 1980 to 2004, the number of published articles was not noticeable, with an average of (18.06 ± 12.82) articles per year, and it rapidly increased in 2012. From 2012 to 2020, the average number of published articles was (182.22 ± 34.58), accounting for 66.76% of the total publications. The majority of articles were published in 2018 (*n* = 221) (Figure 1).

### 3.2 Categorization of Published Articles Based on Country, Region, and Institution

**FIGURE 2:**
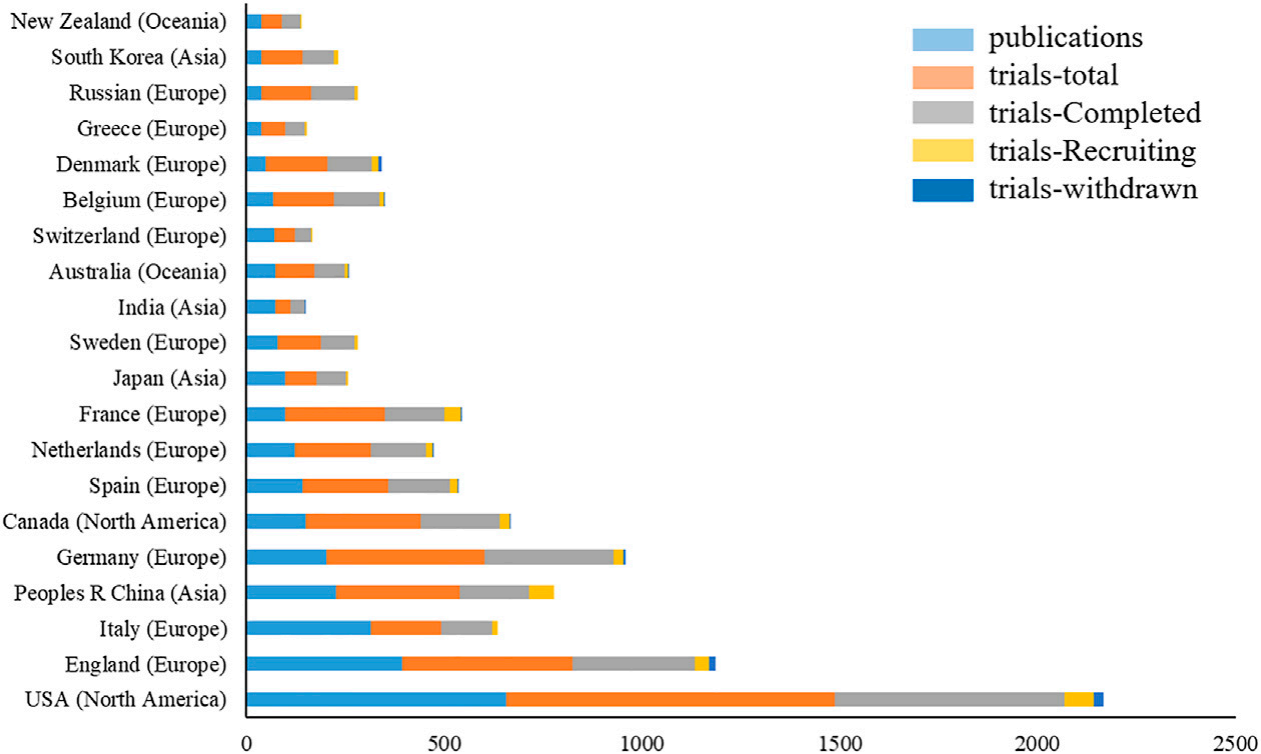
Top 20 countries that published COPD drugs-related studies and the relevant clinical trials from 1980 to 2021.

**FIGURE 3:**
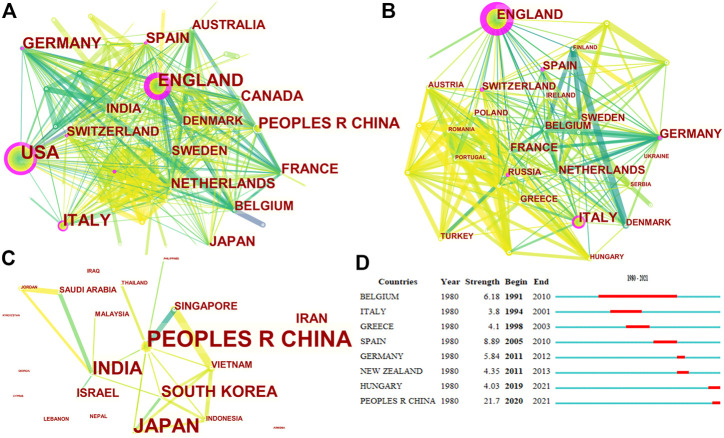
Cooperation among countries that published COPD drugs-related studies from 1980 to 2021. Note: **(A)**: countries around the world; **(B)**: European countries; **(C)**: Asian countries; **(D)**: top 8 countries with the strongest citation bursts.

**FIGURE 4:**
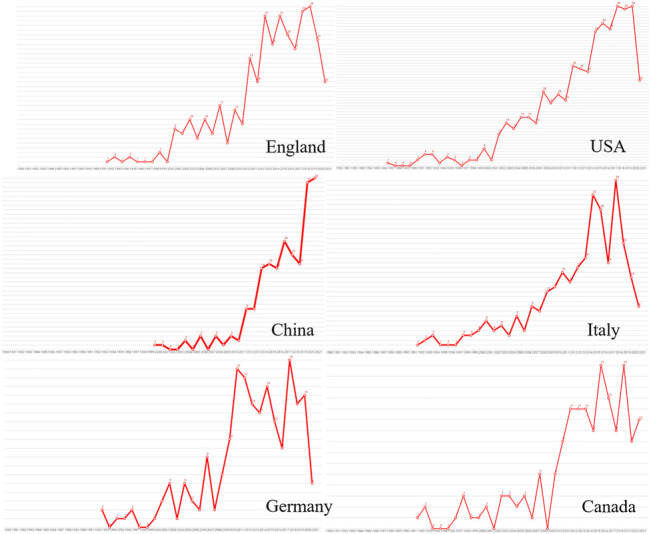
Annual number of COPD drugs-related studies in each country from 1980 to 2021.

**FIGURE 5:**
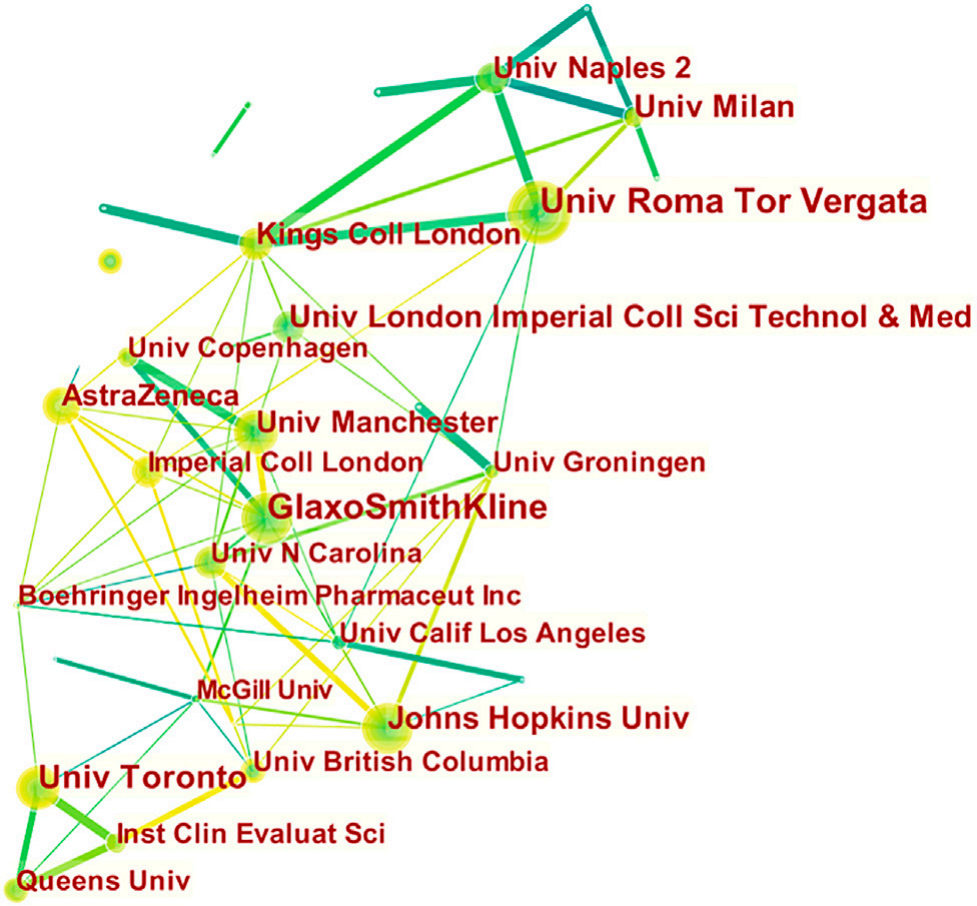
Cooperation among institutions that published COPD drugs-related studies from 1980 to 2021.

**FIGURE 6:**
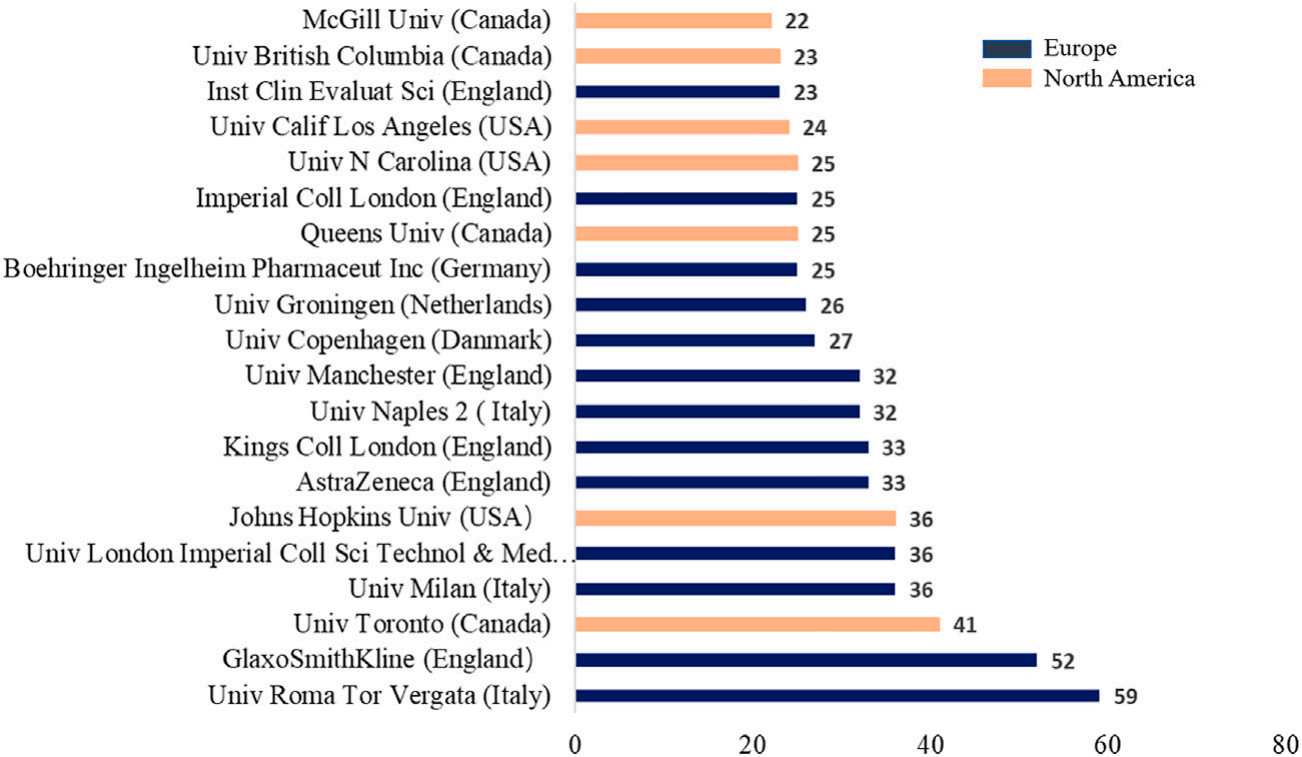
Top 20 institutions that cooperated in publishing COPD drugs-related studies from 1980 to 2021.

Authors were from 83 countries. Among the top 20 countries, including 12 from Europe, 4 from Asia, 2 from North America, and 2 from Oceania. In terms of proportion of each continent, 53.99%, 24.05%, and 15.83% of COPD drugs-related studies were published in Europe, North America, and Asia, respectively (Figure 2). The United States (*n* = 657, centrality = 0.27) and the UK (centrality = 0.41, *n* = 396) accounted for the highest number of articles published. The greatest number of completed clinical trials was recorded in the USA (*n* = 582), followed by Germany (*n* = 327), and the UK (*n* = 308). China (20.13%) has the highest proportion of ongoing clinical trials, followed by France (16.27%), and Greece (13.56%). Compared with Asian countries, a closer cooperation was found among European countries (Figure 3). Among the top 20 research institutions, 13 were located in Europe and 7 in North America. The majority of published articles were from Tor Vergata University of Rome (Italy). Of the top 20 pharmacological companies, GlaxoSmithKline, AstraZeneca, and Boehringer Ingelheim had the highest rates of contribution in the research projects (Figure 4). In terms of the annual number of articles published, among the top 6 countries, the USA ranked the first, and China was the country with a steady increase in the number of published articles (Figures 5, 6). A sudden increase in publications was seen from Univ Roma Tor Vergate, Univ Toronto, Kings Coll London, Johns Hopkins Univ, Univ Manchester, Univ Groningen between 2015 and 2021, Imperial Coll London, Karolinska Inst, Univ Tennessee between 2016 and 2021, and Astrazeneca, Univ Campania Luigi Vanvitelli, German Ctr Lung Res DZL between 2020 and 2021 (Table.1).

**TABLE 1:** Top 27 institutions with the strongest citation bursts that published COPD drugs-related studies from 1980 to 2021.

Begin	End	Strength	Year	Entity
1998	2006	5.3941	1980	A Cardarelli Hosp
2005	2010	4.1669	1980	Univ Calif Los Angeles
2011	2013	4.1035	1980	Univ Auckland
2013	2015	5.8454	1980	Keio Univ
2015	2021	9.4482	1980	Univ Roma Tor Vergata
2015	2019	9.4252	1980	GlaxoSmithKline
2015	2021	9.1131	1980	Univ Toronto
2015	2018	7.3131	1980	Inst Clin Evaluat Sci
2015	2021	6.2404	1980	Kings Coll London
2015	2021	6.1765	1980	Johns Hopkins Univ
2015	2018	5.882	1980	St Michaels Hosp
2015	2021	5.7948	1980	Univ Manchester
2015	2016	5.6786	1980	Univ Naples 2
2015	2019	5.5957	1980	Univ N Carolina
2015	2021	5.2239	1980	Univ Groningen
2015	2016	4.0515	1980	Univ Ferrara
2015	2019	4.0359	1980	Univ Southern Denmark
2016	2021	9.4024	1980	Imperial Coll London
2016	2019	6.5962	1980	Queens Univ
2016	2017	5.3541	1980	GSK
2016	2019	4.4053	1980	Univ Alabama Birmingham
2016	2021	4.0044	1980	Karolinska Inst
2016	2021	3.8956	1980	Univ Tennessee
2017	2021	10.4528	1980	AstraZeneca
2017	2021	9.1418	1980	Univ Campania Luigi Vanvitelli
2017	2019	7.5462	1980	Harvard Med Sch
2017	2021	3.8611	1980	German Ctr Lung Res DZL

### 3.3 Distribution of Fields of Study

**FIGURE 7:**
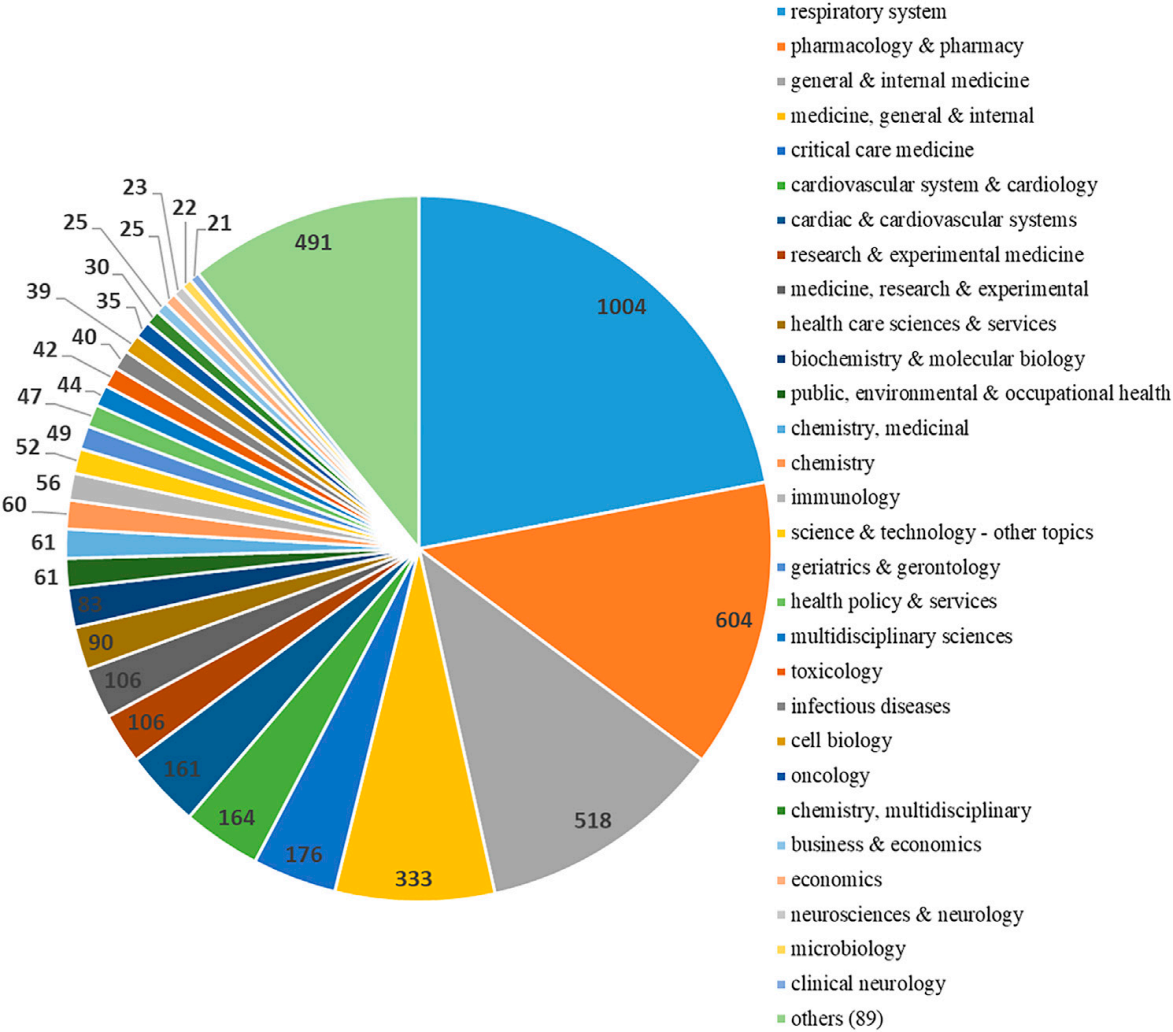
Research areas of COPD drugs-related studies that were published from 1980 to 2021.

The respiratory system (*n* = 1,004), Pharmacology and pharmacy (*n* = 604), and general medicine (*n* = 518) totally accounted for 46.54% of the fields of study. Critical care medicine, Cardiovascular system and cardiology, Biochemistry and molecular biology, Public, environmental and occupational health, Chemistry, medicinal, Immunology, Geriatrics and gerontology, Health policy and services and Toxicology were also included (Figure 7).

### 3.4 Authors’ Collaborations

**FIGURE 8:**
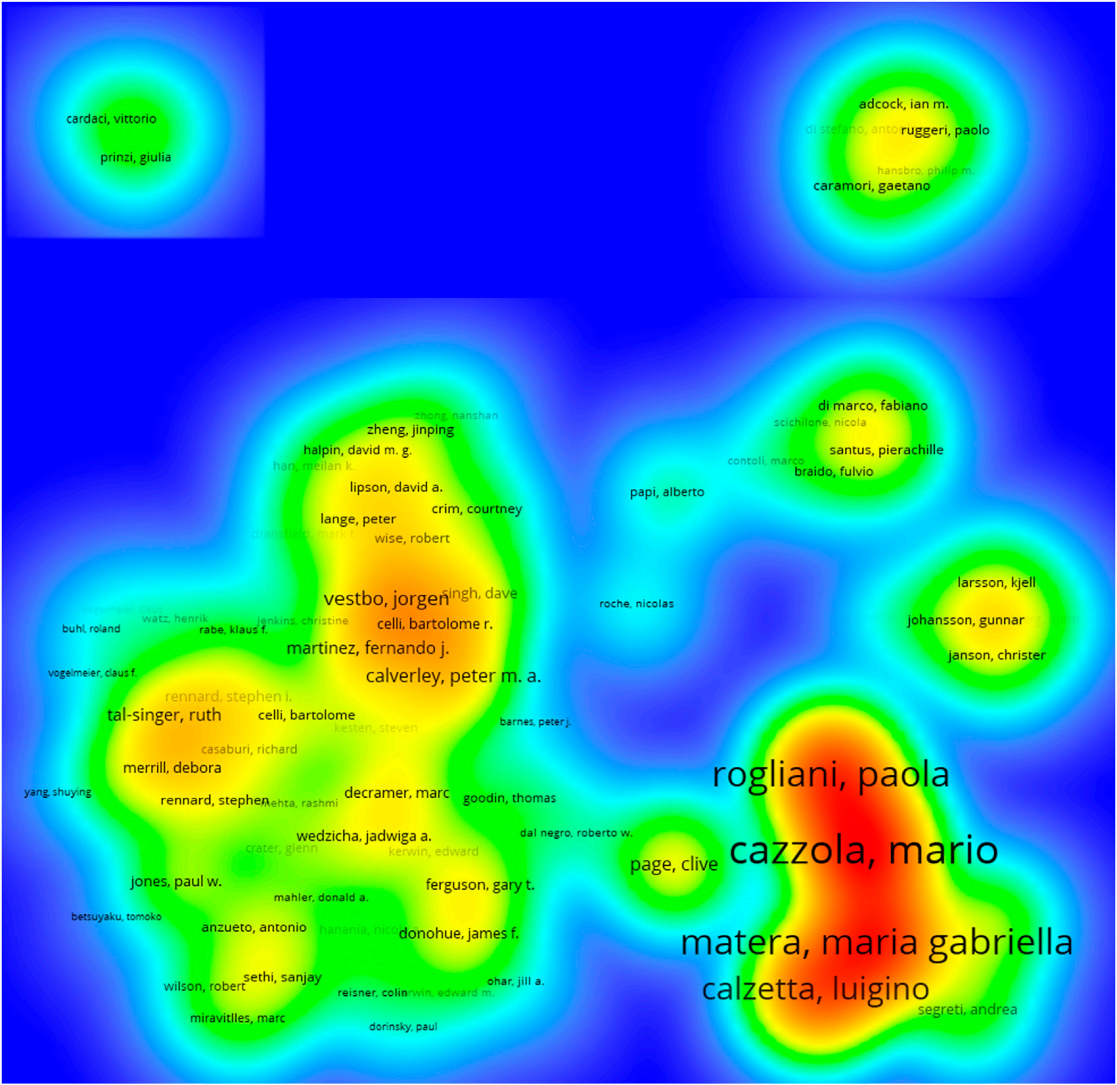
Cooperation among authors that published COPD drugs-related studies from 1980 to 2021.

As displayed in Figure 8, the size of each circle indicates the number of articles produced by the author. The distance between any two circles demonstrates the relatedness of their co-authorship link, and the thickness of the connecting line indicates the strength of the link. We found that authors who published a large number of papers generally had fixed partners, and they accordingly created their own research team.

### 3.5 Citations

#### 3.5.1 Author Co-Citation Analysis

Among the top 10 cited authors (Table 2), Barnes PJ and Celli BR had the highest participation in finalizing global Strategy for the Diagnosis, Management, and Prevention of Chronic Obstructive Lung Disease 2017–2019 Report (GOLD).

**TABLE 2:** The top 10 authors with the highest citations of COPD drugs-related studies from 1980 to 2021.

No	Cited author	Frequency	Author	N
1	Barnes PJ	423	Cazzola M	53
2	Calverley PMA	392	Matera MG	38
3	Celli BR	372	Rogliani P	33
4	Tashkn DP	351	Calzetta L	28
5	Cazzola M	349	Cazzola M	20
6	Vestbo J	342	Vestbo J	16
7	Jones PW	269	Singh D	16
8	Rabe KF	258	Vozoris NT	15
9	Mahler DA	251	Miravitlles M	14
10	Wedzicha JA	244	Donohue JF	14

#### 3.5.2 Journal Co-Citation Analysis

The top 10 cited journals and article published journals are listed in Table 3. The top 3 cited journals were European Respiratory Journal, American Journal of Respiratory and Critical Care Medicine, and Chest. The top 3 journals were International Journal Of Chronic Obstructive Pulmonary Disease, Respiratory Medicine and European Respiratory Journal. The top 10 and 3 cited journals accounted for 42.07% and 17.72% of the total number of COPD-related journals, The top 10 and 3 journals accounted for 25.45% and 12.45% of the total number of COPD-related journals, respectively, indicating that the top journals in the field of respiratory diseases were further concentrated on COPD-related medication.

**TABLE 3:** The list of top 10 cited journals that published COPD drugs-related studies from 1980 to 2021.

Cited-journal	N	Journal	N
Eur Respir J	1,619	Int J Chronic Obstr	111
Am J Resp Crit Care	1,582	Resp Med	108
CHEST	1,562	Eur Respir J	99
Thorax	1,322	CHEST	83
New Engl J Med	1,193	COPD	56
Resp Med	1,103	Pulm Pharmacol Ther	56
Lancet	1,076	Am J Resp Crit Care	38
Resp Res	673	Resp Res	36
Int J Chronic Obstr	621	Cochrane Db Syst Rev	32
COPD	559	Thorax	31

#### 3.5.3 Co-Citation Analysis

**FIGURE 9:**
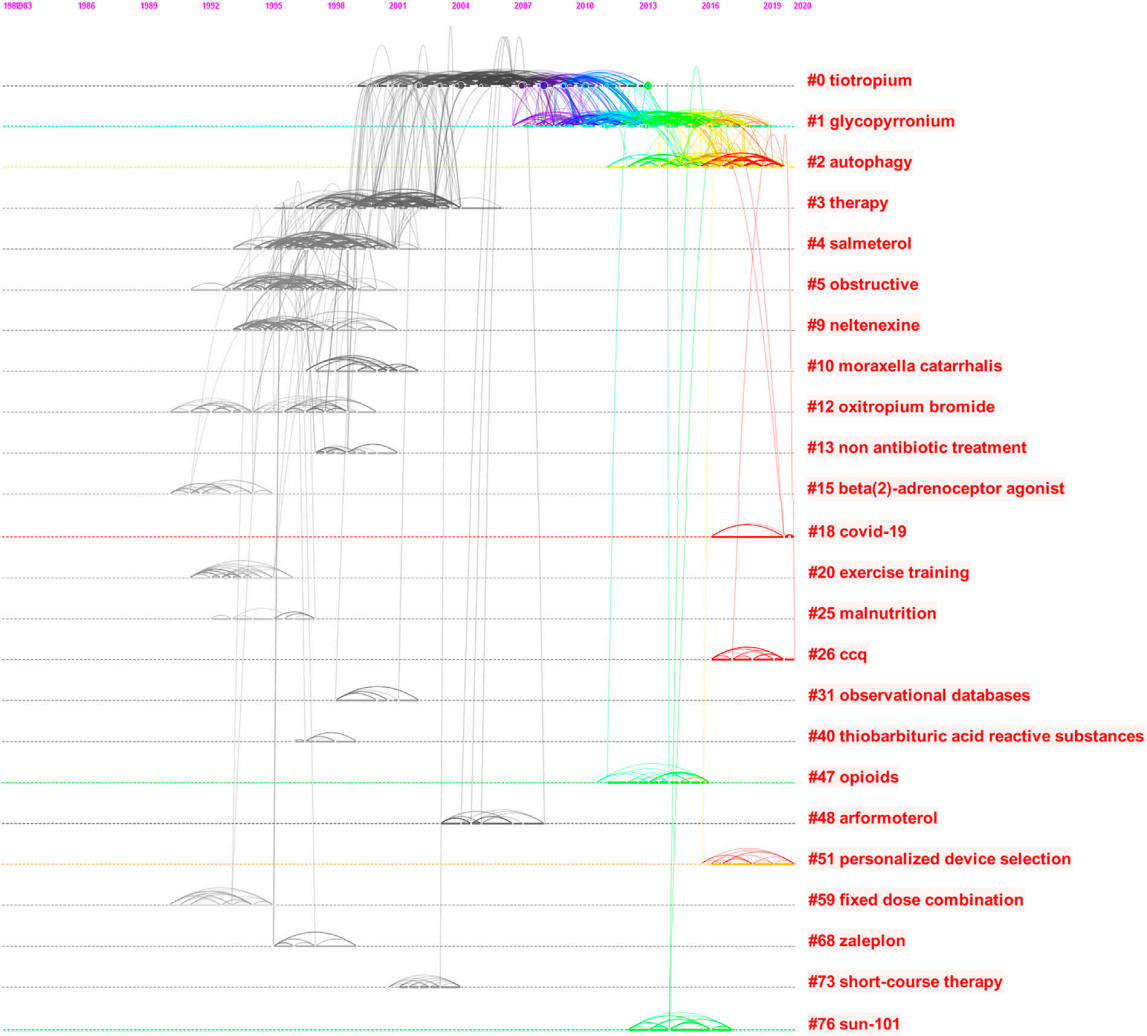
Co-citation analysis of COPD drugs-related studies that were published from 1980 to 2021.

The co-citation analysis showed that, among 2552 articles, 53154 citations were recorded, and the co-citation network is shown in Figure 9. It can be seen that 24 clusters could be achieved, with a Q-value and a silhouette value of 0.902 and 0.954, respectively. The size of the circle represents the size of the surge index. The clusters in the co-citation network are presented in Table 4, including tiotropium, glycopyrronium, salmeterol, neltenexine, and other drugs. The five most frequently cited articles and the most frequently cited researches published in 2021 are listed in Table 5. Research involves Tiotropium, QVA149 [indacaterol/glycopyrronium (Matera et al., 2015)], single-inhaler combination of an extra fine formulation of beclometasone dipropionate, formoterol fumarate, and glycopyrronium bromide and triple therapy (corticosteroid, fluticasone furoate, and vilanterol). The most frequently cited article in 2021 is a real-world clinical evidence.

**TABLE 4:** Co-citation clustering of COPD drugs-related studies that were published from 1980 to 2021.

Cluster	Size	Silhouette	Mean (Year)	Label (LLR)
1	278	0.947	2005	Tiotropium
2	252	0.934	2013	Glycopyrronium
3	173	0.915	2016	Autophagy
4	165	0.945	2000	Therapy
5	138	0.967	1997	Salmeterol
6	126	0.931	1996	Obstructive
7	65	0.988	1996	Neltenexine
8	45	0.995	2000	*Moraxella catarrhalis*
9	40	0.985	1995	Oxitropium bromide
10	34	0.997	1998	Non antibiotic treatment
11	30	1	1991	Beta (2)-adrenoceptor agonist
12	28	1	2019	COVID-19
13	27	0.999	1992	Exercise training
14	22	1	1994	Malnutrition
15	21	1	2018	CCQ
16	20	0.996	2000	Observational databases
17	15	1	1997	Thiobarbituric acid reactive substances
18	14	0.998	2014	Opioids
19	13	0.997	2005	Arformoterol
20	11	1	2017	Personalized device selection
21	10	0.998	1996	Fixed dose combination
22	8	1	1996	Zaleplon
23	7	1	2002	Short-course therapy
24	1	1	2014	Sun-101

**TABLE 5:** The top 5 cited COPD drugs-related studies that were published from 1980 to 2021 and the most cited papers in 2021.

No	Author	Cited frequency	Drug	Condition or disease	Conclusion
1	(Vogelmeier et al., 2011)	455	Tiotropium	Moderate-to-very-severe COPD	In patients with moderate-to-very-severe COPD, tiotropium is more effective than salmeterol in preventing exacerbations
2	(Wedzicha et al., 2013)	364	QVA149	COPD stages III-IV, and one or more moderate COPD exacerbation in the past year	The dual bronchodilator QVA149 was superior in preventing moderate to severe COPD exacerbations compared with glycopyrronium, with concomitant improvements in lung function and health status
3	(Vogelmeier et al., 2013)	238	QVA149	COPD stages II-III, without exacerbations in the previous year	Once-daily QVA149 provides significant, sustained, and clinically meaningful improvements in lung function versus twice-daily salmeterol-fluticasone, with significant symptomatic benefit
4	(Vestbo et al., 2016)	231	Corticosteroid, fluticasone furoate, and vilanterol	Moderate COPD and heightened cardiovascular risk	In patients with moderate COPD and heightened cardiovascular risk, treatment with fluticasone furoate and vilanterol did not affect mortality or cardiovascular outcomes, reduced exacerbations, and was well tolerated
5	(Singh et al., 2016)	216	Single-inhaler combination of an extra fine formulation of beclometasone dipropionate, formoterol fumarate, and glycopyrronium bromide (BDP/FF/GB)	COPD had post-bronchodilator FEV1 of lower than 50%, one or more moderate-to-severe COPD exacerbation in the previous 12 months, CAT ≥10, and a Baseline Dyspnea Index focal score of 10 or less	This paper provide evidence for the clinical benefits of stepping up patients with COPD from an inhaled corticosteroid/long-acting β2-agonist combination treatment to triple therapy using a single inhaler
6	(Izquierdo et al., 2021)	13	Clinical Management of COPD in a Real-World Setting	COPD	This study identifies the main features of an unselected COPD population and the major errors made in the management of the disease

### 3.6 Analysis of Keywords

**FIGURE 10:**
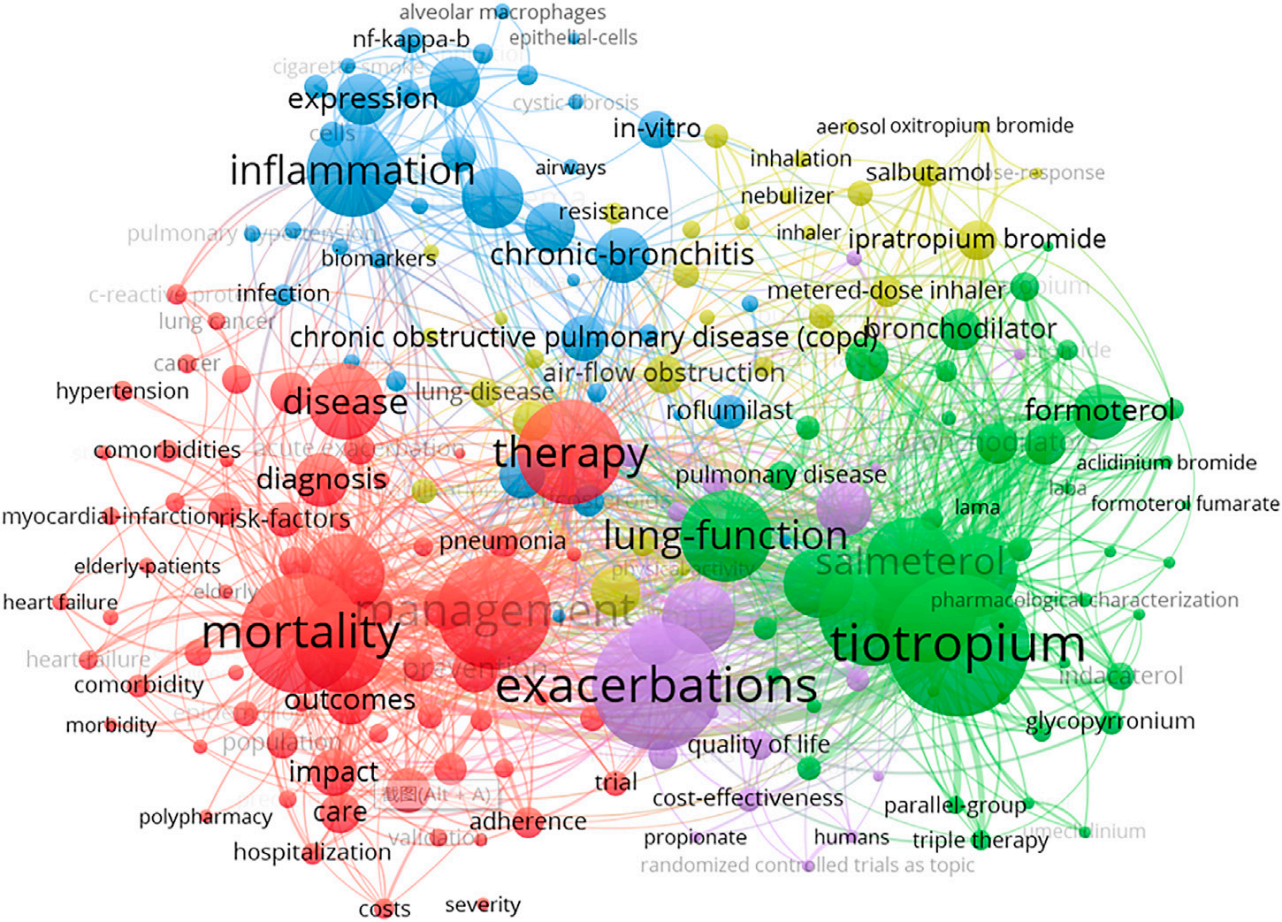
Keywords analysis of COPD drugs-related studies that were published from 1980 to 2021.

**FIGURE 11:**
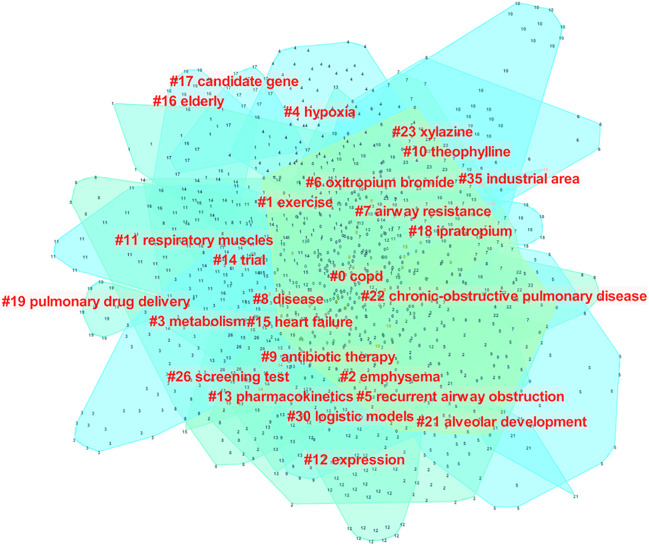
Keywords co-occurrence analysis of COPD drugs-related studies that were published from 1980 to 2021.

When the keywords with the same meaning were combined, the frequency of the published keywords was statistically analyzed, and the top 20 keywords are summarized in Table 6. A total of 26 items could be achieved by cluster analysis (Table 7), including interventions, pathogenesis of the disease, complications, etc. Interventions for COPD included exercise, Oxitropium bromide, antibiotic therapy, theophylline, ipratropium. The pathogenesis of the disease included metabolic pathways, hypoxia, recurrent airway obstruction, airway resistance, respiratory muscles, alveolar development and other factors. Heart failure was the major complication. Susceptible groups of people (e.g., elderly) and different risk factors (e.g., living in industrial areas) were recorded as well. In addition to the disease name, the most frequently cited keywords were related to exacerbation, tiotropium, mortality, management, therapy, double blind and efficacy, reflecting the importance of medications in the management of COPD, prevention of acute attacks, and reduction of mortality (Figures 10, 11).

**TABLE 6:** The top 20 keywords of COPD drugs-related studies that were published from 1980 to 2021.

Rank	Frequency	Centrality	Key words
1	1,312	0.04	COPD
2	717	0.03	Obstructive pulmonary disease
3	286	0.02	Exacerbation
4	244	0.03	Tiotropium
5	206	0.06	Mortality
6	192	0.05	Management
7	176	0.03	Therapy
8	173	0.05	Double blind
9	170	0.12	Efficacy
10	169	0.01	Lung function
11	155	0.02	Inflammation
12	147	0.02	Risk
13	143	0.01	Salmeterol
14	136	0.01	Safety
15	131	0.11	Disease
16	131	0.05	Prevalence
17	115	0.03	Bronchodilator
18	105	0.03	Quality of life
19	102	0.04	Chronic bronchiti
20	101	0.03	Acute exacerbation

**TABLE 7:** Clustering of keywords in COPD drugs-related studies that were published from 1980 to 2021.

Cluster	Size	Silhouette	Mean (Year)	Label (LLR)
0	203	0.564	2002	COPD
1	94	0.824	1997	Exercise
2	85	0.902	2000	Emphysema
3	76	0.901	1998	Metabolism
4	71	0.921	1996	Hypoxia
5	62	0.939	1998	Recurrent airway obstruction
6	58	0.861	1996	Oxitropium bromide
7	58	0.879	2000	Airway resistance
8	58	0.938	1997	Disease
9	56	0.896	2000	Antibiotic therapy
10	55	0.866	2000	Theophylline
11	54	0.937	1998	Respiratory muscles
12	46	0.939	1999	Expression
13	43	0.934	1996	Pharmacokinetics
14	33	0.907	2000	Trial
15	31	0.962	2000	Heart failure
16	20	0.958	1997	Elderly
17	16	0.999	1999	Candidate gene
18	14	0.986	2000	Ipratropium
19	10	0.999	2002	Pulmonary drug delivery
21	10	0.992	2001	Alveolar development
22	9	0.998	2003	COPD
23	8	0.996	1998	Xylazine
26	7	1	1991	Screening test
30	4	0.995	2004	Logistic models
35	3	1	2000	Industrial area

So-called “burst words” represent words that are cited frequently over a period of time, (Liang et al., 2017) Burst keywords show the frontier topics and key areas of research. This article focuses on the keywords with the strongest citation bursts that continue to 2021, keywords suddenly increased: “Safety” suddenly increase between 2014 and 2021. “risk,” “oxidative stress,” “risk factor” suddenly increase between 2015 and 2021. “Drug delivery” suddenly increase between 2016 and 2021. “impact,” “device” suddenly increase between 2017 and 2021. “Prevalence,” “triple therapy,” “prevention,” “parallel group,” “health,” “comorbidity” suddenly increase between 2018 and 2021. “adherence,” “*in vitro*,” “association,” “resistance,” “nf kappa b,” “COPD exacerbation,” “drug,” “inhaler,” “tuberculosis,” “metered dose inhaler” suddenly increase between 2019 and 2021. “COPD,” “depression,” “burden,” “COVID-19,” “intervention,” “diagnosis,” “older adult” suddenly increase between 2020 and 2021 (Table 8).

**TABLE 8:** Keywords with the strongest citation bursts of COPD drugs-related studies from 1980 to 2021.

Begin	End	Strength	Year	Entity
2014	2021	8.30	1980	Safety
2015	2021	12.16	1980	Risk
2015	2021	7.98	1980	Oxidative stress
2015	2021	5.36	1980	Risk factor
2016	2021	10.72	1980	Drug delivery
2017	2021	10.65	1980	Impact
2017	2021	5.31	1980	Device
2018	2021	14.09	1980	Prevalence
2018	2021	10.64	1980	Triple therapy
2018	2021	9.60	1980	Prevention
2018	2021	8.40	1980	parallel group
2018	2021	8.08	1980	Health
2018	2021	6.50	1980	Comorbidity
2019	2021	8.98	1980	Adherence
2019	2021	8.58	1980	*In vitro*
2019	2021	6.96	1980	Association
2019	2021	6.55	1980	Resistance
2019	2021	5.92	1980	nf kappa b
2019	2021	5.12	1980	Copd exacerbation
2019	2021	4.92	1980	Drug
2019	2021	4.82	1980	Inhaler
2019	2021	4.18	1980	Tuberculosis
2019	2021	4.01	1980	Metered dose inhaler
2020	2021	7.93	1980	COPD
2020	2021	6.18	1980	Depression
2020	2021	5.67	1980	Burden
2020	2021	5.38	1980	COVID-19
2020	2021	5.28	1980	Intervention
2020	2021	5.12	1980	Diagnosis
2020	2021	3.77	1980	Older adult

## 4 Discussion

In recent years, significant progress has been made in the development of new pharmacological and surgical tools to treat COPD, while the rates of prevalence and mortality owing to COPD are still noticeable. Through the visual bibliometric analysis by the CiteSpace software, we could understand the progression of research on COPD drugs, enabling us to more accurately predict the study direction in the future. The number of publications can reflect the overall development trend. The number of COPD drugs-related articles has been elevated from 1980 to 2021, especially an explosive growth was recorded in 2012, indicating that the treatment of COPD has markedly attracted clinicians’ attention in the recent decades.

From the perspective of categorization of published articles based on country, the USA is the country with the largest number of published articles and completed clinical trials, highlighting the important role of this country in the treatment of COPD. However, in terms of the proportion of ongoing clinical trials, China has the highest proportion, suggesting that China will play a more pivotal role in the medication of COPD in the future. A sudden increase in publications was seen from Hungary between 2019 and 2021, and China between 2020 and 2021 also proved this. From the perspective of cooperation among countries, the cooperation among European countries was closer than that among Asian countries. In the recent three decades, the top 20 institutions, with a particular concentration on the treatment of COPD, were from North America (*n* = 7) and Europe (*n* = 13). The identification of core journals with high publication and co-citation counts provide important information for authors to select high-quality journals (Wang et al., 2020). The present study revealed that a variety of journals have published COPD-drugs related articles. High cited journals were relatively concentrated, and the top 10 journals accounted for 42.07% of the total number of journals related to the medication of COPD, suggesting that the importance of COPD was acknowledged by these journals.

The co-citation analysis showed that, among 2552 articles, 53154 citations were recorded, and the co-citation network indicated that 24 clusters could be achieved, with a Q-value and a silhouette value of 0.902 and 0.954, respectively. Bronchodilators play a pivotal role in the treatment of symptomatic patients with COPD. Inhaled short-acting bronchodilators are currently recommended for relieving symptoms of patients with mild COPD, whereas inhaled long-acting bronchodilators are recommended as first-line agents for maintenance therapy of patients with moderate and severe COPD (Steiropoulos et al., 2012). Tiotropium resulted in a higher FEV1 than placebo at 24 months and ameliorated the annual decline in the FEV1 after bronchodilator use in patients with COPD of GOLD stage 1 or 2 (Zhou et al., 2017). This also reveals the significant role of drug delivery systems, for example, SUN-101 is a combination of glycopyrrolate delivered through an innovative, electronic nebulizer (Kerwin et al., 2017). Meanwhile, fixed-dose combination and personalized medicine are also important topics. One study (Hu et al., 2020) showed that acute exacerbation and hospitalization of COPD patients were infrequent during the coronavirus disease 2019 (COVID-19) pandemic. However, COVID-19 patients with pre-existing COPD had a higher risk of all-cause mortality.

Based on the keyword analysis of COPD-drugs related articles, we summarize the following four keywords: 1) Management: the target of COPD management is to improve a patient’s functional status and quality of life by preserving optimal lung function, improving symptoms, and preventing the recurrence of exacerbations. 2) Anticholinergics, such as ipratropium bromide and tiotropium bromide, are the most effective group of bronchodilators in the treatment of COPD. Tiotropium bromide was the first long-acting muscarinic antagonist (LAMA) available for COPD in clinical practice. There are two pulmonary drug delivery modes: delivery of inhalation powder via a dry powder inhaler (DPI) and drug delivery via a soft mist inhaler (SMI). Tiotropium was found comparable to inhaled corticosteroid (ICS)/long-acting β2-agonist (LABA) in improving lung function and reducing exacerbations and had a greater influence on exacerbation rates than LABAs. Hence, fixed-dose LAMA/LABA combinations have also been developed. Studies showed that tiotropium and olodaterol dual bronchodilator therapy may improve lung function and quality of life and reduce exacerbations in patients with COPD in early stages (Criner and Duffy, 2021). The co-formulation of indacaterol and glycopyrronium can be usefully utilized to optimize and maximize bronchodilation in many COPD patients, who do not experience an adequate airflow increase by using a single bronchodilator (Pelaia et al., 2014). Oxitropium bromide can improve the exercise capacity of patients with stable COPD (Ikeda et al., 1994). Our results showed that, tiotropium has not only attracted clinicians’ attention in the treatment of COPD previously, but also it plays a significant role in the development of further effective therapies for COPD. 3) Pulmonary drug delivery is a compelling noninvasive technique to deliver systemic drugs into circulation. Nowadays, most inhaled drugs are delivered by pressurized metered dose inhaler, (Newman and Dhand, 2015) dry powder inhaler (Hickey and Dhand, 2015) or nebulizer (Fink et al., 2015). The advantage of COPD pulmonary drug delivery is to use a relatively low dose, a low incidence of systemic side effects and a rapid onset of action. And pulmonary drug delivery is a very complicated process. First, the respiratory tract has evolved defense mechanisms that are intended to keep inhaled materials out of the lungs, as well as removing or inactivating them once they have been deposited (Labiris and Dolovich, 2003). Second, it is necessary for a patient to use an inhaler device, and to use it correctly (Newman, 2014). Handling errors of inhaler devices are underestimated in real life and are associated with an increased rate of severe COPD exacerbation (Molimard et al., 2017). Meeting the challenge of delivering drugs to the lungs requires selection of an appropriate inhaler and formulation (Newman, 2017). A review article on tiotropium/olodaterol in the treatment of COPD showed that once-daily delivery of fixed-dose combinations of tiotropium and olodaterol via a very efficient and simple-to-use inhaler device such as Respimat significantly contributes to enhance the therapeutic efficacy of dual bronchodilation, as well as to increase patient adherence to inhaled treatment (Pelaia et al., 2015). Therefore, it is highly essential to further concentrate on the development of COPD drugs in terms of components of drug, drug delivery systems, as well as training patients to take medications safely. 4) COPD in elderly patients: Khakban A predict, the total number of patients diagnosed with COPD will increase by 155%, and COPD-related hospitalization will increase by 210% in 2010–2030. By 2030, 55% of the patients with COPD will be 75 years and older. (Khakban et al., 2017) Frontier studies on the treatment of COPD should particularly involve elderly populations. COPD is a common disease among elderly patients, in which treatment of elderly patients with COPD is highly challenging, and randomized controlled trials may underestimate the risk of adverse effects of interventions. Although age is an important factor in the incidence of COPD, there is a lack of age-based research on COPD drugs. This may be one of the next efforts in COPD drug research.

Diagnosis and treatment of COPD are complicated because COPD may manifest as multiple phenotypes. Research showed that the phenotypical approach is crucial in the management of COPD as it allows to individualize the therapeutic strategy and to obtain more effective clinical outcomes (Dal Negro et al., 2021). According to the analysis of keywords, we summarize five research directions in the following. 1) Identification of the risk factors of COPD: exposure to cigarette smoke worsens lung edema and inflammation, (Hou et al., 2019) and smoking is the main risk factor for COPD (Mannino and Buist, 2007). However, there are still numerous unknown risk factors for COPD, which should be explored through epidemiological assessment. 2) Triple therapy: research showed that addition of fluticasone-salmeterol to tiotropium therapy did not statistically influence rates of COPD exacerbation but did improve lung function, quality of life, and hospitalization rates in patients with moderate to severe COPD (Aaron et al., 2007). Subsequent studies of this kind have gradually become the hotspot and frontier of COPD drug research. The route of administration should be mainly pulmonary inhalation, and attention should be paid to the development and utilization of drug delivery devices. Study showed that, once-daily fluticasone furoate/umeclidinium/vilanterol (FF/UMEC/VI) was non-inferior to twice-daily budesonide/formoterol via metered-dose inhaler plus once-daily tiotropium via HandiHaler (BUD/FOR + TIO) for weighted mean change from baseline in 0–24 h FEV1 at Week 12 in patients with COPD. Greater improvements in trough and serial FEV1 measurements at Week 12 with FF/UMEC/VI versus BUD/FOR + TIO, together with similar health status improvements and safety outcomes including the incidence of pneumonia (Ferguson et al., 2020). And for patients with frequent and/or severe acute exacerbations in the past, although these patients have received triple, LABA/ICS, single bronchodilator or double bronchodilator, they used closed triple inhalation therapy compared with fixed-dose double bronchodilator therapy, can still benefit from mortality (GOLD, 2021). The main administration route should be pulmonary inhalation, and further attention should be paid to the research and development of drug delivery systems. 3) COPD and comorbidity: the Global Initiative for GOLD guidelines (2011) has recommended that, in general, the presence of co-morbidities should not alter COPD treatment, and comorbidities should be treated urgently, and a new version has emphasized the role of acute exacerbation and complications of COPD in the disease assessment. The most common comorbidities are ischaemic heart disease, diabetes, skeletal muscle wasting, cachexia, osteoporosis, depression, and lung cancer (Corlateanu et al., 2016). It was found that, the relationship between complications of COPD and treatment is still in infancy, and depression and COVID-19 are worthy of consideration to better explore the mentioned relationship. 4) Elderly patients with COPD: further research should be conducted to improve medications more effectively for elderly patients with COPD. 5) Pharmacoeconomics analysis: pharmacoeconomics analysis of COPD better clarifies the economic benefits of the proposed medications. Revealing the appropriate initial and maintenance drug regimens for patients with different phenotypes or subtypes of COPD, which is expected to control medical costs. Study showed that promptly initiating triple therapy after two moderate or one severe exacerbation is associated with decreased morbidity and economic burden in COPD, (Tkacz et al., 2022) for example.

The two important limitations of the current study should be pointed out. Firstly, we only analyzed studies indexed in the WOSCC database. Secondly, the Matthew effect, which might influence the results of bibliometric analysis, was not considered (Jiang et al., 2021).

In summary, the administration of bronchodilators and pulmonary drug delivery systems, as well as consideration of elderly COPD patients require further attention in frontier studies on medications for COPD, and exact mechanisms underlying the pathogenesis of COPD should be explored as well.
